# *Artemisia* dominant species succession relating to the soil moisture decrease in abandoned land of the Loess Plateau (China): comparative study of drought-adaptive characteristics

**DOI:** 10.1186/s40064-016-2678-3

**Published:** 2016-07-07

**Authors:** Yong Wang, Jing Yu, Pengguo Xia, Shaoxuan He, Ziyun Zhou, Ruilian Han, Zongsuo Liang

**Affiliations:** Institute of Soil and Water Conservation, Chinese Academy of Sciences and Ministry of Water Resources, Yangling, 712100 China; College of Horticulture and Landscape, Hainan University, Haikou, 570228 China; College of Life Science, Zhejiang Sci-Tech University, Hangzhou, 310018 China; College of Life Science, Northwest A&F University, Yangling, 712100 China; University of Chinese Academy of Sciences, Beijing, 100049 China

**Keywords:** The Loess Plateau, *Artemisia*, Antioxidative system, Drought adaptation, Anatomical characteristics

## Abstract

*Artemisia scoparia*, *Artemisia sacrorum* and *Artemisiagiraldii* were three dominant *Artemisia* species which successive grew in the secondary succession on abandoned land of the Loess Plateau. The succession accompanied the soil moisture steady decrease with field age after their abandonment. To elucidate the relationship between the *Artemisia* species succession and their drought-adaptation, three dominant species and a contrastive species *Artemisia annua* (mesophyte), were selected to compare their drought-resistant characteristics, including morphological and anatomical traits of leaf and root. Then physiological responses were investigated in mature plants after drought treatment. The results indicated that three dominant species leaf presented drought-adaptive structures, such as bushy trichomes, transitional or isolateral leaf cells, thick cuticles and epidermal cells. However, *A. annua* had no leaf traits involved in drought-adaptation. In addition, *A. sacrorum* and *A. giraldii* contained large root systems, while *A. scoparia* and *A. annua* utilized succulent roots. The physiological responses to drought suggested that *A. giraldii* had strong regulation in water using strategy, growth, as well as superoxide dismutase and catalase activity. *A. sacrorum* and *A. giraldii* could maintain high ascorbate peroxidase activity and malondialdehyde content, while *A. scoparia* and *A. giraldii* presented higher peroxidase activity, ascorbate and soluble sugar content. *A. annua* exhibited high proline and carotenoid contents under drought. The drought-resistant of the four *Artemisia* species presented the order of *A. giraldii* > *A. sacrorum* > *A. scoparia* > *A. annua*, which was consistent with their succession on abandoned land.

## Background

The Loess Plateau of China, one of the best-known areas in the world, is characterized by drought-prone climate with infrequent precipitation and high levels of soil erosion (Douglas 1989; Liu et al. 2012; Shi and Shao 2000). Water shortage is a major limiting factor for vegetation restoration in the loess hilly region of northwest China (Chen et al. 2007). In particular, it more serious in the abandoned land during natural vegetation recovery processes. However, after land closure, native species, such as *Artemisia* (family *Compositae*) (Jiang et al. 2013), rapidly occupied the abandoned land, and exhibited strong adaptive-ability to the drought environment during secondary succession (An and Shangguan 2010; Wang et al. 2010; Zhang et al. 2000; Zhang 2005). As pioneers, *Artemisia scoparia* Waldst. et Kit., *Artemisia sacrorum* Web. ex Stechm. and *Artemisia giraldii* Pamp. are three dominant species in the secondary vegetation succession on abandoned land (Du et al. 2007, 2013 Zhang et al. 2006; Zhao et al. 2014; Zhou et al. 2006). *A. scoparia* is annual or biennial species that acted as pioneers and strongly dominated the early stages (1–6 years) of succession (Jiao et al. 2005; Wang 2003). Then, *A. sacrorum*, a perennial species, had its peak abundance at intermediate stages (15–35 years) of succession (Wang 2003). *A. giraldii* is also a perennial species, appeared at mid-succession stage and gradually increased in abundance during succession, becoming dominant at late stages (25–46 years) (Du et al. 2007; Jiao et al. 2005). *A. sacrorum* and *A. giraldii* could grow together and form a community at mid-succession stage of succession (Zhao et al. 2014). Some investigation revealed that soil moisture decreased steadily with field age after their abandonment in the loess hilly region (Jiao et al. 2005; Wang 2003; Du et al. 2007; Wang et al. 2009). In this regard, we hypothesize that these *Artemisia* species have different characters and strategies in relation to drought-adaptation. These differences could illustrate the succession of three *Artemisia* species on their abandoned land due to the soil moisture decrease.

Plants can avoid or resist drought through multiple strategies and coordination mechanisms. To maintain tissue water balance under arid environment, plants can minimize water loss by flexible stomatal adjustment, drought-adaptive leaf anatomical structure and reduction of the growth of the aboveground, as well as maximize water supply by the large and/or succulent root system of the underground (Bosabalidis and Kofidis 2002; Flexas and Medrano 2002; Hanba et al. 2000; Leal-Bertioli et al. 2012; Kozlowski and Pallardy 2002). Apart from these morphological changes, tissue antioxidative system is important for plants resisting drought stress (Chaves et al. 2003; Mittler 2002), which include non-enzymes antioxidants (e.g. ascorbate, glutathione, and tocopherol) and antioxidative enzymes (e.g., superoxide dismutase, peroxidase, and catalase). Another potentially important mechanism of drought tolerance in cells is osmotic adjustment (Seki et al. 2007), which is achieved from the accumulation of compatible solutes, e.g. proline, betaine, soluble sugar, and sugar alcohol, in protoplasm to maintain cell turgor during drought stress (Ashraf and Foolad 2007; Carillo et al. 2011; Hasegawa 2013; Szabados and Savoure 2010).

In this study, we compared the morphological and anatomical traits of leaf and root in three *Artemisia* species. Then, we planted these species in pots of glasshouse to investigate the physiological response to soil drought. To better understand the drought-adaptive traits of three dominant species, *Artemisia annua* as contrastive species was selected from the loess hilly, which is a common annual or perennial mesophyte living in areas with adequate soil water content (Chen et al. 1993). According this research, it well elucidated that the primary principles of *Artemisia* species changed with the secondary succession on the Loess Plateau.

## Methods

### Plant materials and treatments

The seeds of three *Artemisia* species were collected from the natural communities of abandoned lands in October 2013, and naturally dried them in the laboratory. Simultaneously, mature seeds of *A. annua* were collected in the same region with sufficient soil moisture conditions. The abandoned lands were located in Gaoqiao Country (36°39′N, 109°11′E), Shaanxi Province, which belongs to a typical hilly region of Loess Plateau. Parent rock of the study areas is Loess soil, which contains poor nutrient amounts and water conservation. The average altitude is 1800 m. The annual average temperature can range from 7 to 11 °C, and the lowest and highest temperature can reach −10 and 25 °C, respectively. Annual precipitation is invariably lower than 500 mm, and most of the precipitation is concentrated from July to September (Liu et al. 2012; Du et al. 2007). The age of the abandonment lands were determined from interviews with local farmers. To obtain the morphological and anatomical parameters of all species, their seeds were sown in the field of the Institute of Soil and Water Conservation, Chinese Academy of Sciences and Ministry of Water Resources (ISWC, CAS and MWR). All plants were grown at the same conditions for one year, and the mature plants were used for investigation.

To obtain the physiological responses profiling to drought, four species mature plants were subjected to drought treatment using pot culture in the greenhouse of ISWC, CAS and MWR. During the subsequent spring, the seeds of each species were sown in 20 pots (27 cm deep and 35 cm in diameter) respectively, 80 pots in total. Each pot contained 13 kg soil, which was the native abandoned land, air dried, sieved (0.5 mm), and evenly mixed. The field capacity water content (FC) was 22.5 % (weight basis). Water was added into the bottom of pot through a PVC pipe (1.5 cm in diameter) to control soil moisture. After 4 months growing, final 6 mature plants with similar sizes in each pot were chosen, and two different watering regimes were applied to each species over the following one month (August–September 2014). For each species, one group of ten pots was used as controls (CK), in which plants were watered every 2–3 days to maintain their soil water content at 75 % FC; another group (WS) of ten pots of each species was subjected to drought stress and experienced sustained water supply (soil water maintained at 35 % FC). Each treatment of each species included three replicates, with three pots per replicate. To control soil water content in the pots, we re-watered the pots to different FCs by replacing the amount of water transpired daily, as determined by an electronic weighing scale (*d* = 0.001 kg). After 1 month of different irrigation treatments, healthy, mature leaves were harvested for physiological analysis.

### Sample preparation for scanning electron microscopy (SEM)

For analysis of leaf epidermis and attachment, mature leaf fragments (2–4 mm^2^) of four *Artemisia* species from the field were immediately fixed in the field in 2.5 % glutaraldehyde in 0.1 M phosphate buffer (pH 7.4) overnight at 4 °C. Samples for SEM were prepared by the method of Pathan et al. (2008). Features observed on the abaxial and adaxial surfaces, including stomata density (NE, number of stomata mm^−2^), epidermal cell density (CE, number of epidermal cells mm^−2^), and trichomes, were recorded. The numbers of epidermal cells and stomata were determined through six observations of each surface with a 0.33 mm^2^ area of each of the leaflets of five plants to determine stomatal density. Stomatic index (IE) was calculated as:

$${\text{IE}} = 100 \times\left[ {{\text{NE}}/\left( {{\text{CE}} + {\text{NE}}} \right)} \right],$$

where NE corresponds to the stomatal density and CE signifies the epidermal cell density (Cutter 1987).

### Sample preparation for leaf tissue anatomy analysis

For analysis of leaf tissue anatomical characteristic, leaf fragments (1–2 mm^2^) were excised from the mid-blade of mature leaves of four *Artemisia* species from the field. Pre-fixation was performed similar to the sample preparation for SEM, whereas post-fixation was conducted in 1 % osmium tetroxide in phosphate buffer. Following ethanol dehydration, the material was embedded in epoxy resin. Semi-thin sections (0.5–1 μm) were cut using a Leica EM UC7 ultramicrotome and collected onto glass slides. The sections were stained with toluidine blue (5 min) and observed under Nikon E800 microscope. The thicknesses (μm) of the leaves, epidermis, palisade parenchyma, spongy parenchyma, and cuticles were measured using the measurement tool of the microscope imaging system.

### Root morphology and anatomy analysis

At least five plants of each *Artemisia* species were carefully removed from the soil. The roots were washed carefully. The lengths of the main root, lateral root and the diameter at the base of the main root, were determined using a Vernier caliper. Lateral root number was counted, and root volume was determined through the water replacement method.

For analysis of root tissue anatomical characteristic, five root samples (0.5 cm × 0.5 cm piece) of each species were collected and immediately fixed in the field in formaicacetic acid-alcohol (FAA). Root cross sections were obtained according to the method described by Cutter (1987). Five replications were prepared for each species. The cross sections were observed under an optical microscope.

### Leaf relative water content (RWC), biomass and root/shoot ratio

For RWC assay, after fresh weight determination, the samples were floated on de-ionized water for 6 h under low light radiation to saturate the leaf and then quickly blot dried prior to the determination of turgid weight. The dry weight of leaves was determined after oven-drying at 70 °C for 72 h. RWC was calculated according to Smart and Bingham (1974) and using the following formula:

$${\text{RWC }}\left( \% \right) = 100\times\left( {{\text{fresh weight}} - {\text{dry weight}}} \right)/\left( {{\text{turgid weight}} - {\text{dry weight}}} \right).$$

To determine the biomass and root/shoot ratio, plants from each treatment were randomly selected, and shoots and roots were separated at the end of the drought treatment. Then, the fresh weights of shoots and roots were recorded, and the samples were oven-dried at 70 °C for 72 h and then weighed.

### Gas exchange measurements

Net CO_2_ assimilation (*A*), transpiration (*E*), stomatal conductance (*Gs*) and intercellular CO_2_ concentration (*C*_*i*_) were measured with a portable photosynthesis open system (ADC BioScientific LCpro + System, England) at midmorning (10:00 AM – 11:00 AM) after 1 month of different irrigation treatments. At least six mature and healthy leaves from each treatment were used to measure these parameters at saturating light (1300 μmol m^−2^ s^−1^), 25 °C, and CO_2_ concentration (*C*_*a*_) 370 ± 8 μmol^−1^. Intrinsic water use efficiency (*WUEi*) was calculated as the ratio of *A/E*, while the stomatal limitation (*Ls*) was obtained by the formula:

$$Ls = \, 1 \, {-}C_{i} /C_{a} .$$

### Lipid peroxidation and leaf enzyme assays

Fresh mature leaf samples were frozen in liquid nitrogen immediately after harvesting and stored at −80 °C until lipid peroxidation and enzyme assays. Lipid peroxidation was determined by estimating the malondialdehyde (MDA) content in 1 g leaf fresh weight according to Hodges et al. (1999). Leaves enzymes were extracted according to An et al. (2013) with some modifications. Random 0.2 g fresh samples was homogenized with 4 mL 50 mM Na phosphate buffer solution (pH 7.8) including 1 % PVP and 0.1 mM EDTA, and centrifuged at 12,000×*g* for 20 min at 4 °C. The supernatant was used for measuring the following enzymes and protein content assays.

Superoxide dismutase (SOD; EC 1.15.1.1) activity was determined by the method of Giannoplitis and Ries (1977) by measuring the inhibition in the photochemical reduction of nitroblue tetrazolium (NBT) spectrophotometrically at 560 nm. One unit of SOD activity was defined as the quantity of SOD required to produce a 50 % inhibition of NBT and the specific enzyme activity was expressed as U g^−1^ dry weight h^−1^. Catalase (CAT; EC 1.11.1.6) activity assay was measured according to Aebi (1984) which measures the decline of the extinction of H_2_O_2_ (extinction coefficient 0.04 mM^−1^ cm^−1^) at the maximum absorption at 240 nm for 3 min. Peroxidase (POX; EC 1.11.1.7) activity was done according to the method of An et al. (2013). The assay depends on the increase in absorbance at 470 nm, by the rate of formation of the oxidized guaiacol. One unit of POX activity was defined as the amount of enzyme that made A470 increase 0.01 per min mg^−1^ protein. The enzyme activity was expressed in terms of U g^−1^ dry weight min^−1^. Ascorbate peroxidase (APX; EC 1.11.1.11) was based upon the method of Chen and Asada (1989), which was recorded as the decrease in absorbance at 290 nm for 3 min as ascorbate was oxidized and calculated from the extinction coefficient (ε) 2.8 mM^−1^ cm^−1^.

### Proline, soluble sugar, ascorbic acid (ASA) and carotenoid

Free proline content was extracted from 0.2 g of dry leaf samples in 3 % (w/v) aqueous sulfosalicylic acid and measured using the ninhydrin reagent according to a previously described method of Bates et al. (1973). Absorbance of the fraction with toluene aspired from the liquid phase was determined at 520 nm. Proline concentration was determined using the calibration curve and expressed as μmol proline g^−1^ DW.

Soluble sugars were extracted by heating the samples in 80 % ethanol for 30 min three times. After centrifugation for 10 min at 5000×*g*, the supernatant were used for analysis. Total soluble sugar content was determined using the supernatant through the classical anthrone method (Yemm and Willis 1954).

ASA content was analyzed using a previously described method by Arakawa et al. (1981) with some modifications. Fresh leaf segments (0.5 *g*) were homogenized in 10 mL of 5 % trichloroacetic acid and centrifuged at 12,000×*g* for 10 min. ASA content was measured based on the reduction of ferric ions to ferrous ions with ASA in acid solution and the formation of red chelate between ferrous ions and bathophenanthroline, with absorbance at 530 nm.

Carotenoid contents were measured using a previously described method by Lichtenthaler (1987) with some modifications. Fresh mature leaf samples (0.1 g) were cut and immersed in 80 % cold acetone for 3 days in the dark at 4 °C. Absorbance was recorded at 645, 663 and 470 nm.

### Statistical analysis

All analyses were performed on a completely randomized design, and data obtained was subjected to one-way analysis of variance (ANOVA) to highlight differences in relevant variables. The “Tukey HSD” post hoc multiple comparison tests were used for comparison of treatment means at *P* < 0.05. Data were analyzed using SPSS software (SPSS 17.0, SPSS Inc., USA). In all figures the spread of values is shown as error bars representing standard errors of the means.

## Results

### Leaf epidermis hair in the four *Artemisia* species

**Fig. 1:**
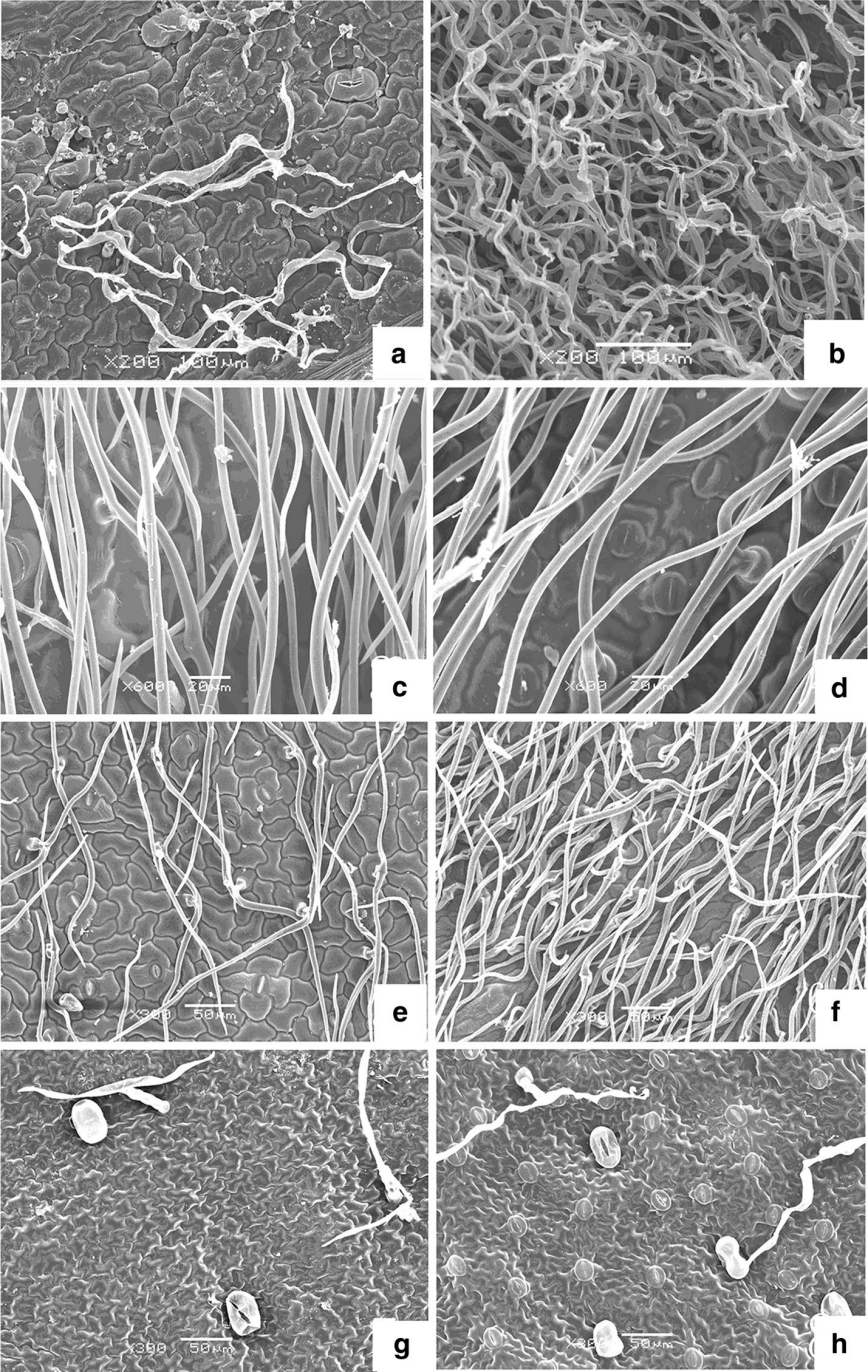
Leaf epidermis of the four *Artemisia* species. **a** Upper epidermis of *A. sacrorum*; **b** lower epidermis of *A. sacrorum*; **c** upper epidermis of *A. scoparia*; **d** lower epidermis of *A. scoparia*; **e** upper epidermis of *A. giraldii*; **f** lower epidermis of *A. giraldii*; **g** upper epidermis of *A. annua*; and **h** lower epidermis of *A. annua*

As shown in Fig. 1, epidermal hair was found on both leaf surfaces of the four *Artemisia* species. *A. scoparia* and *A. giraldii* contained similar trichomes, which appeared as two forks parallel to both surfaces, whereas the upper-trichomes of *A. giraldii* were sparse. *A. sacrorum* possessed bushy leaf epidermal hair only on the lower surface. Both surfaces of *A. annua* and the upper-surface of *A. sacrorum* presented very few trichomes.

**Fig. 2:**
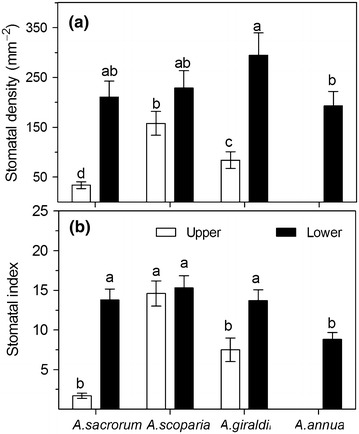
Stomatal density (**a**) and stomatal index (**b**) of the four *Artemisia* species. Upper leaf epidermis of *A. annua* is without stomatal distribution. *Different letters* indicate significant differences at *P* < 0.05. *Values* are presented as the mean ± SD, *N* = 6

Stomata were distributed on both surfaces of the leaves of *A. sacrorum*, *A. scoparia*, and *A. giraldii* but only on the lower-surface of *A. annua* leaves. *A. giraldii* presented the highest stomatal density on the lower-surface over the three other species, whose stomatal density was not significantly different (Fig. 2a). However, stomatal density significantly differed on the upper-surface among *A. sacrorum*, *A. scoparia*, and *A. giraldii* (*P* < 0.05). The stomatal index of the upper-surface showed the same conditions in terms of stomatal density and presented the order of *A. scoparia* > *A. giraldii* > *A. sacrorum*. *A. annua* presented the lowest stomatal index of the lower-surface over *A. scoparia*, *A. giraldii*, and *A. sacrorum*, whose index was not significantly different (Fig. 2b). The stomatal density and stomatal index of the lower-surface were higher than that of the upper-surface in all species, except *A. scoparia*.

### Leaf anatomy analysis in the four *Artemisia* species

**Fig. 3:**
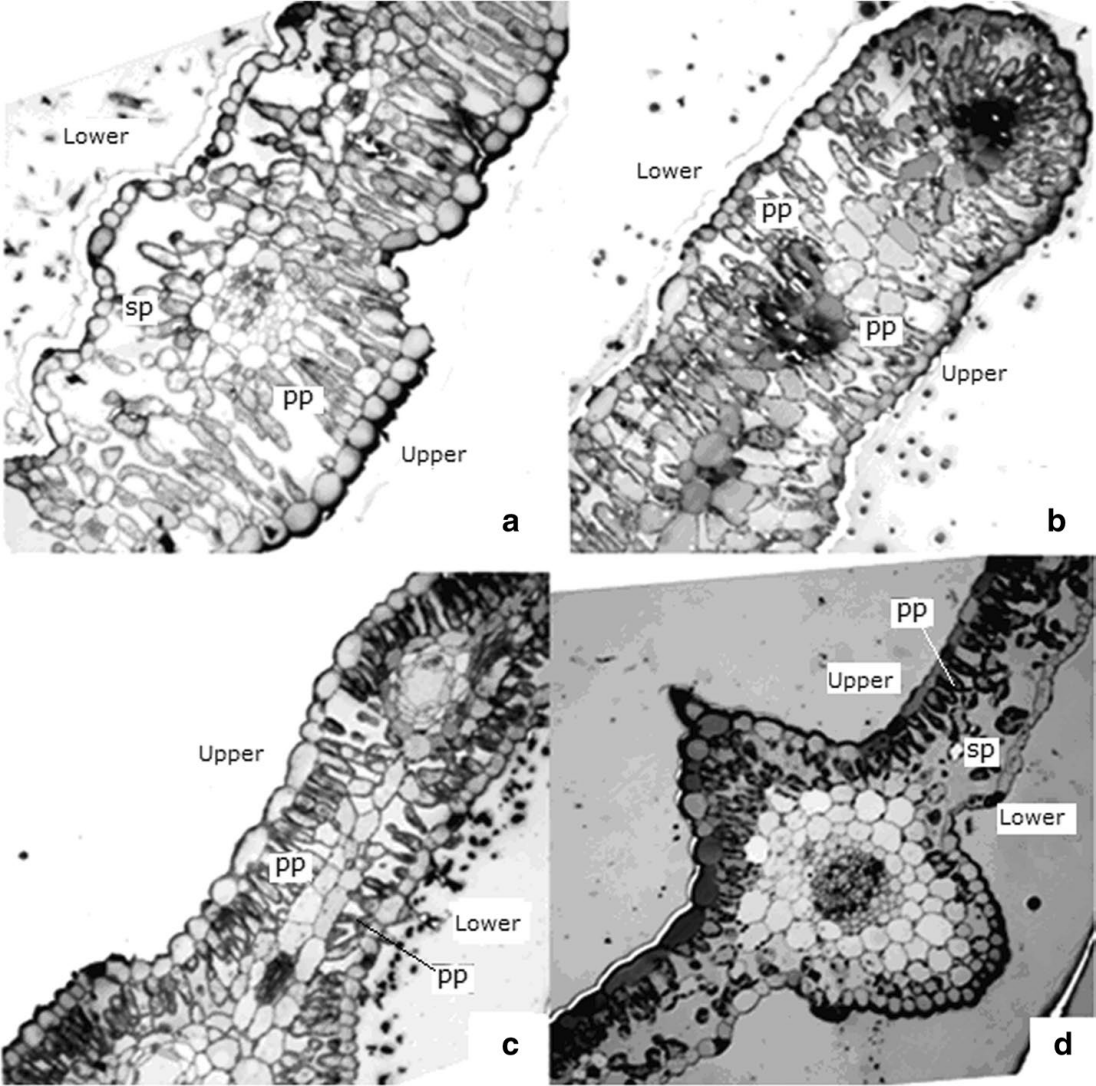
Leaf cross sections (×100) of the *A. sacrorum* (**a**), *A. scoparia* (**b**), *A. giraldii* (**c**) and *A. annua* (**d**). *Both upper* and *lower* palisade parenchyma was found in *A. scoparia* (**b**) and *A. giraldii* (**c**), whereas only *upper* palisade parenchyma was observed in *A. sacrorum* (**a**) and *A. annua* (**a**). *PP* palisade parenchyma, *SP* spongy parenchyma

As shown in Table 1 and Fig. 3, *A. scoparia* presented the thickest blade, whereas *A. annua* exhibited the thinnest blade, with approximately half of the leaf thickness of *A. sacrorum* and *A. scoparia*. *A. sacrorum* demonstrated the highest epidermal thickness, thickness of palisade parenchyma and cuticles. Except the maximum leaf thickness among the four species, *A. scoparia* showed the maximum thickness of lower palisade and spongy parenchyma, as well as TPP %. *A. giraldii* possessed median levels in all indices. *A. annua* presented the lowest values in almost all of the indices, except for TSP %, compared with the other species.

**Table 1:** Comparison of anatomical features of the leaves of the four *Artemisia* species

	*A. sacrorum*	*A. scoparia*	*A. giraldii*	*A. annua*
Types of leaf cells	Transitional form	Isolateral leaf	Isolateral leaf	Bifacial leaf
Leaf thickness (μm)	189.8 ± 1.3^b^	206.0 ± 2.1^a^	136.7 ± 1.5^c^	90.6 ± 1.0^d^
Epidermal thickness (μm)				
Upper	23.6 ± 0.8^a^	17.2 ± 0.9^b^	16.6 ± 0.8^b^	12.7 ± 1.3^c^
Lower	15.9 ± 1.4^a^	15.7 ± 0.4^a^	13.8 ± 0.6^b^	11.6 ± 0.9^c^
Thickness of palisade parenchyma (μm)				
Upper	77.1 ± 0.5^a^	60.2 ± 0.1^b^	38.4 ± 0.9^c^	28.0 ± 0.5^d^
Lower	–	47.6 ± 0.4^a^	27.8 ± 1.1^c^	–
Thickness of spongy parenchyma (μm)	60.9 ± 1.7^a^	65.5 ± 1.5^a^	40.0 ± 0.8^b^	31.0 ± 0.9^c^
TPP/TSP	1: 0.79 ± 0.02^b^	1:0.61 ± 0.05^a^	1:0.60 ± 0.04^a^	1:1.11 ± 0.02^c^
Thickness of cuticles (μm)				
Upper	7.5 ± 0.9^a^	3.7 ± 0.7^b^	3.7 ± 0.4^b^	3.1 ± 0.3^bc^
Lower	3.3 ± 0.3^a^	2.9 ± 0.5^ab^	2.8 ± 0.4^ab^	2.7 ± 0.2^b^
TPP %	40.1 ± 1.1 %^c^	52.6 ± 1.1 %^a^	49.4 ± 0.8 %^ab^	29.6 ± 1.5 %^d^
TSP %	32.0 ± 1.2 %^b^	32.5 ± 0.8 %^b^	30.2 ± 0.9 %^b^	38.6 ± 1.0 %^a^

Values are presented as mean ± SD, *N* = 6. Different letters in the same row indicate significant difference at *P* < 0.01

*TPP* thickness of palisade parenchyma, *TSP* thickness of spongy parenchyma

### Root morphology and anatomy of the four *Artemisia* species

**Fig. 4:**
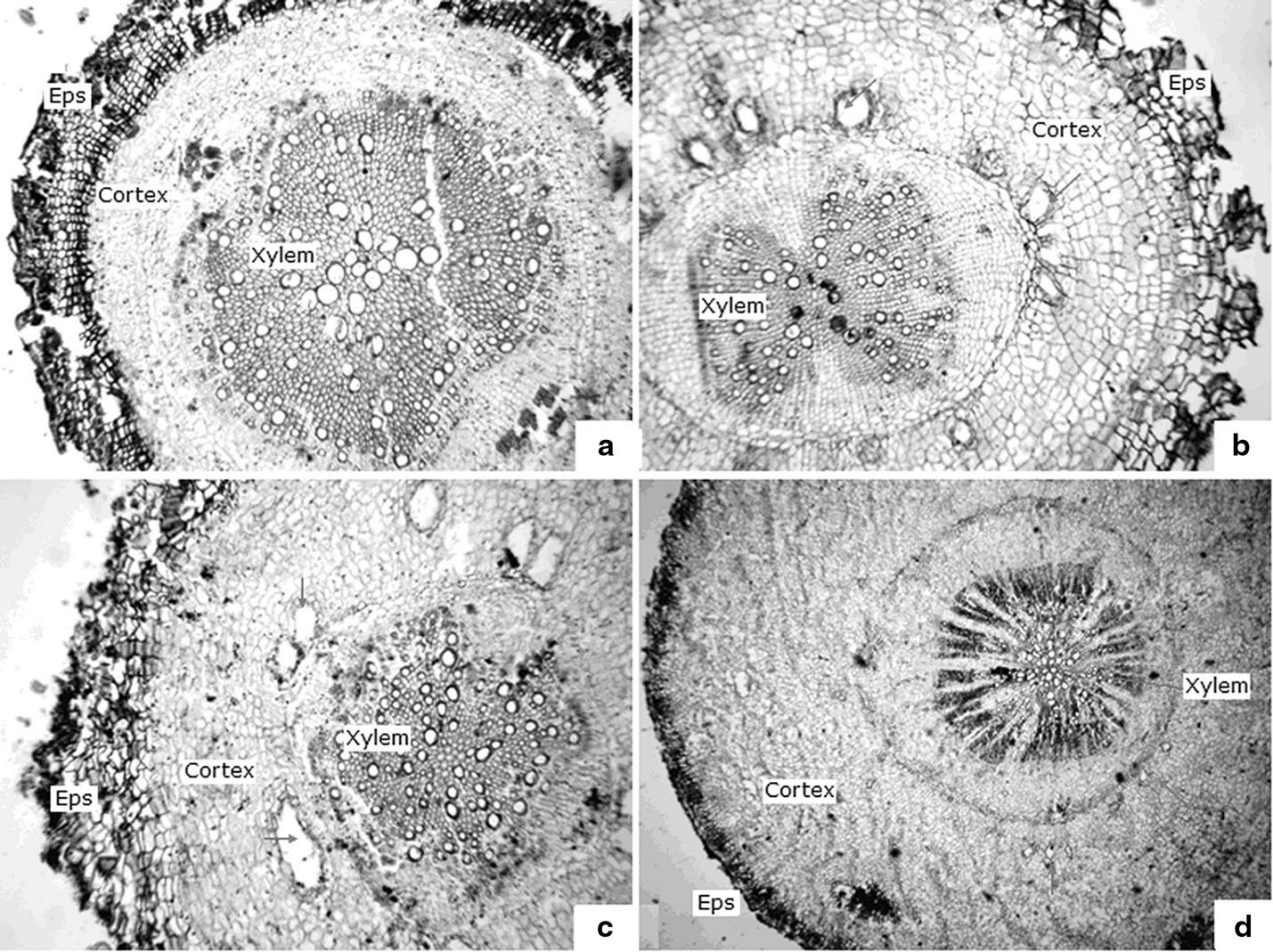
Root cross sections of the *A. sacrorum* (**a**, ×100), *A. scoparia* (**b**, ×100), *A. giraldii* (**c**, ×100) and *A. annua* (**d**, ×50). Large proportion of xylem was observed in *A. sacrorum* (**a**), whereas large proportion of cortex was observed in *A. annua* (**d**)

As shown in Table 2, *A. sacrorum* and *A. giraldii* presented a relative thin and long root system pattern, with large root volume and numerous lateral roots. By contrast, the root system of *A. scoparia* and *A. annua* was relative thick and short, with low root volume and few lateral roots. Thus, the order of roots’ dry biomasses was as follows: *A. giraldii* > *A. sacrorum* > *A. scoparia* > *A. annua*. The root anatomy showed relative thicker epidermis and numerous xylem vessels in *A. giraldii*, *A. sacrorum* and *A. scoparia*. However, *A. giraldii*, *A. scoparia*, and *A. annua* possessed numerous parenchyma cells in the root cortex and several holes in the cortex layer (Fig. 4).

**Table 2:** The root morphology parameters of the four *Artemisia* species

Species	Root dry weight (g plant^−1^)	Main root length (cm)	Lateral root length (cm)	Lateral root number	Diameter of root stalk (cm)	Root volume (cm^3^)
*A. sacrorum*	4.52 ± 0.17^b^	34.57 ± 3.30^a^	35.34 ± 2.00^a^	18.00 ± 2.00^a^	0.58 ± 0.06^c^	10.86 ± 1.12^a^
*A. scoparia*	2.71 ± 0.23^c^	11.00 ± 1.58^c^	10.63 ± 1.99^b^	12.60 ± 3.10^b^	1.14 ± 0.13^a^	5.63 ± 0.98^b^
*A. giraldii*	5.61 ± 0.10^a^	31.30 ± 2.65^a^	32.16 ± 2.89^a^	18.80 ± 2.6^a^	0.58 ± 0.04^c^	10.00 ± 1.23^a^
*A. annua*	1.13 ± 0.09^d^	25.50 ± 1.65^b^	9.11 ± 1.23^b^	13.00 ± 2.89^b^	0.86 ± 0.10^b^	3.08 ± 0.79^c^

Values are presented as mean ± SD, *N* = 6. Different letters in the same column indicate significant difference at *P* < 0.01

### RWC, biomass and root/shoot ratio

**Fig. 5:**
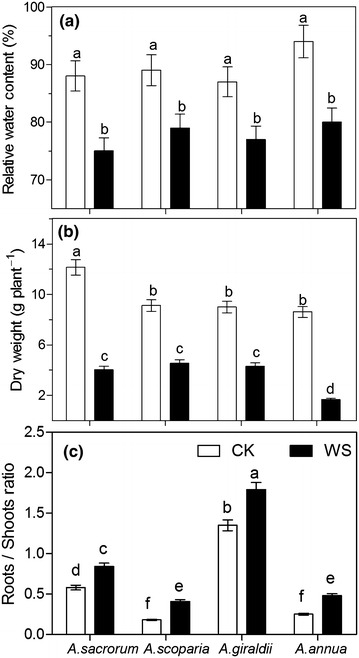
Relative water content (**a**), biomass (**b**) and root/shoot ratio (**c**) of four *Artemisia* species in one growing season. *CK* control setup, *DW* drought stress. *Different letters* indicate significant difference at *P* < 0.05. *Values* are presented as mean ± SD, *N* = 6

As shown in Fig. 5, drought markedly decreased the RWC of four *Artemisia* species. *A. sacrorum*, *A. scoparia*, *A. giraldii* and *A. annua* were decreased by 14.7 11.2, 11.6, and 15.7 % respectively. Drought significantly decreased dry weight of the four *Artemisia* species. Dry weight reduced by 67, 50, 52, and 70 %, in *A. sacrorum*, *A. scoparia*, *A. giraldii*, and *A. annua*, respectively. Root/shoot ratios considerably increased by 44.8, 128, 32.6, and 92 % in *A. sacrorum*, *A. scoparia*, *A. giraldii*, and *A. annua*, compared with the control levels, respectively. *A. sacrorum* and *A. giraldii* exhibited higher root/shoot ratios than *A. scoparia* and *A. annua*.

### Leaf gas exchange parameters

**Fig. 6:**
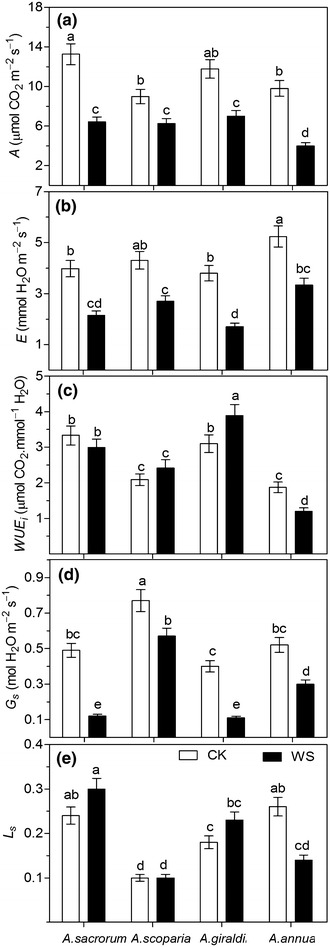
Photosynthetic parameters of four *Artemisia* species subjected to soil drought stress. *A*, Net photosynthetic rate (**a**); *E*, transpiration rate (**b**); *WUEi*, instantaneous water use efficiency (**c**); *Gs*, stomatal conductance (**d**); *Ls*, stomatal limitation (**e**). *CK* control setup, *DW* drought stress. *Different letters* indicate significant difference at *P* < 0.05. *Values* are presented as mean ± SD, *N* = 6

As shown in Fig. 6, significant differences were found among four *Artemisia* species with respect to the physiological variable studied. Under different drought condition, the highest inhibition of leaf photosynthesis (*A*) was found in *A. annua*, followed by *A. sacrorum*, *A. giraldii* and *A. scoparia*, whereas on significant difference of *A* was found among three dominant species. Drought markedly decreased the transpiration rate (*E*) in four species, and it presented the highest inhibition in *A. giraldii* (55.3 %), followed by *A. sacrorum* (46.1 %), *A. annua* (36.4 %) and *A. scoparia* (32.7 %). *A. giraldii* and *A. sacrorum* showed higher intrinsic water use efficiency (*WUEi*) than *A. annua* and *A. scoparia* under control condition. Under drought condition, *A. giraldii* presented enhanced *WUEi* by 25.6 %, whereas *A. annua* showed decreased *WUEi* by 35.9 %. *A. scoparia* was the highest stomatal conductance (*Gs*) specie under control or drought condition. Both *A. sacrorum* and *A. giraldii* showed the lower *Gs* under drought stress. With respect to the stomatal limitation, only *A. annua* was declined by drought stress.

### Lipid peroxidation and leaf antioxidative enzyme assays

**Fig. 7:**
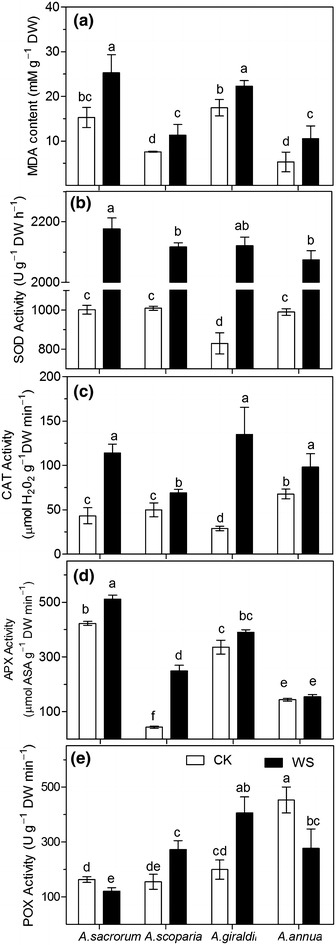
Lipid peroxidation (**a**) and leaf antioxidative enzyme activity (**b**–**e**) of the four *Artemisia* species subjected to soil drought stress. *CK* control setup, *DW* drought stress. *Different letters* indicate significant difference at *P* < 0.05. *Values* are presented as mean ± SD, *N* = 6

As shown in Fig. 7a, drought significantly increased malondialdehyde (MDA) content in the leaves of the four *Artemisia* species. *A. annua* showed the maximum increase in MDA content (98 %), whereas *A. giraldii* showed the minimum increase in MDA content (27 %). The MDA contents of *A. giraldii* and *A. sacrorum* were higher than those of *A. scoparia* and *A. annua* in both the constitutive and stressed groups.

Drought significantly activated superoxide dismutase (SOD) and catalase (CAT) in the leaves of the *Artemisia* plants (Fig. 7b, c). *A. giraldii* exhibited the greatest change in activities of SOD (155.4 %) and CAT (365.7 %) under drought conditions, whereas *A. scoparia* and *A. annua* showed the lower increase in SOD (109.8 and 109.4 %, respectively) and CAT activities (37.9 % and 44.7 %, respectively). Drought markedly increased ascorbate peroxidase (APX) activity in the leaves of *A. sacrorum* (21.5 %) and *A. scoparia* (472.6 %). However, APX activity in *A. giraldii* and *A. sacrorum* were higher than that in *A. scoparia* and *A. annua* in both the constitutive- and stressed-groups.

Under control conditions, the POX activity in *A. annua* was higher than that in the other three species (Fig. 7e). Under drought conditions, POX activity increased in *A. scoparia* and *A. giraldii* by 75.8 and 102.7 %, respectively, whereas decreased in *A. sacrorum* and *A. annua* by 25.7 and 38.7 %, respectively.

### Proline, soluble sugar, ascorbic acid (ASA) and carotenoid

**Fig. 8:**
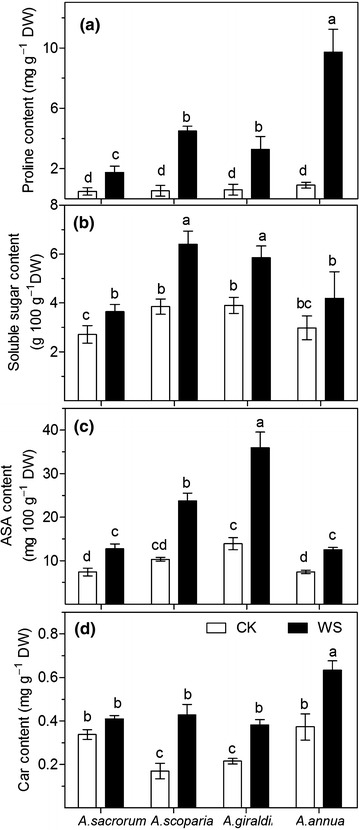
Proline (**a**), soluble sugar (**b**), ascorbic acid (**c**) and carotenoid contents (**d**) in the leaves of the four *Artemisia* species. *CK* control setup, *DW* drought stress. *Different letters* indicate significant differences at *P* < 0.05. *Values* are presented as mean ± SD, *N* = 6

Proline content was not significantly different among the four species under control conditions (Fig. 8a). Compared with the control levels, proline content significantly increased by 9.8, 7.4, 2.6 and 4.4 times in *A. annua*, *A. scoparia*, *A. sacrorum* and *A. giraldii* respectively, under drought conditions. By contrast, *A. scoparia* and *A. giraldii* showed higher increase in soluble sugar content (66 and 61 %, respectively) than *A. sacrorum* and *A. annua* (40 and 34 %, respectively) under drought conditions (Fig. 8b). ASA content considerably increased by 130, 158, 72 and 68 % in *A. scoparia*, *A. giraldii*, *A. sacrorum* and *A. annua*, respectively, respectively, under drought conditions when compared with the control levels (Fig. 8c). Drought significantly increased pigment contents carotenoid, in the leaves of the four species (Fig. 8d). *A. sacrorum* and *A. annua* showed higher carotenoid contents than *A. scoparia* and *A. giraldii*. However, under drought conditions, *A. scoparia* and *A. annua* exhibited greater increase than *A. sacrorum* and *A. giraldii* in carotenoid content.

### Principal component analysis (PCA) of the physiological characteristics in the four *Artemisia* species

**Fig. 9:**
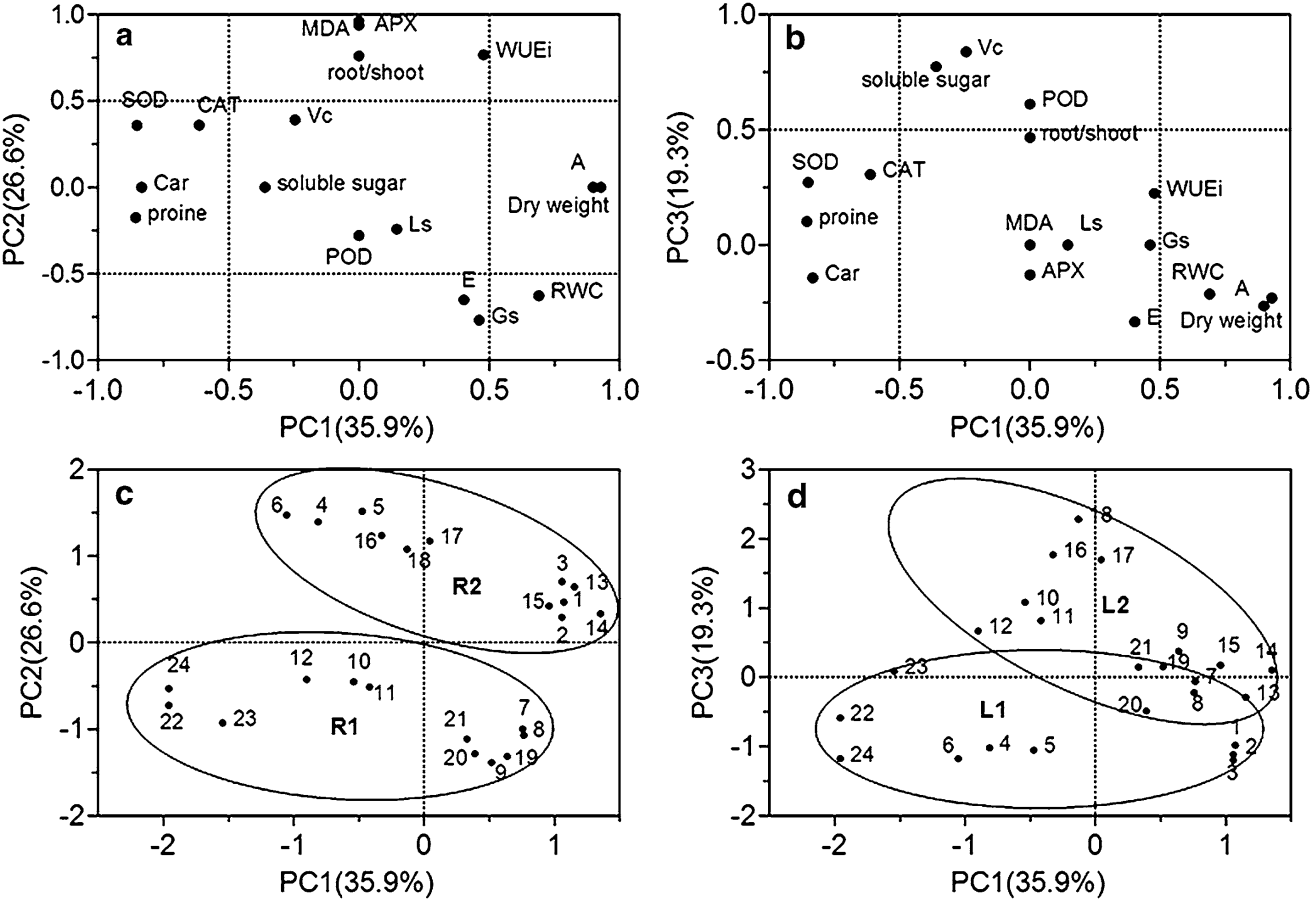
PCA (Principal component analysis) of physiological changes of 4 species. **a**, **b** PC 1–PC 2 and PC 1–PC 3 variables loading plots; **c**, **d** PC 1–PC 2 and PC 1–PC 3 samples score plots. PC 1 mainly including dry weight, photosynthetic rate (*A*), relative water content (RWC), CAT, SOD, Car, proline; PC 2 mainly include APX, MDA, root/shoot, instantaneous water use efficiency (*WUEi*), stomatal conductance (*Gs*) and transpiration rate (*E*); PC 3 mainly include ASA, POD and soluble sugar. *R 1*: the small root system, *R 2*: the large root system, *L 1*: isolateral leaf or transitional form, *L 2*: bifacial leaf. *1–3*: *A. sacrorum* in control, *4–6*: *A. sacrorum* in drought stress, *7–9*: *A. scoparia* in control, *10–12*: *A. scoparia* in drought stress, *13–15*: *A. giraldii* in control, *16–18*: *A. giraldii* in drought stress, *19–21*: *A. annua* in control, *22–24*: *A. annua* in drought stress

PCA was used to evaluate the changes of tissue water condition, growth, antioxidants and osmotic substance under drought condition. Three principal components (PC 1, PC 2 and PC 3) were extracted, and these dimensions explained over 81.8 % of the total variability (Fig. 9). The data dimensions were therefore reduced from 17 to 3 for further data processing.

PC1 was heavily and positively associated with dry weight, photosynthetic rate (*A*), RWC, CAT, SOD, Car, praline, and PC 2 gave a high weighting to APX, MDA, root/shoot, instantaneous water use efficiency (*WUEi*), stomatal conductance (*Gs*) and transpiration rate (*E*) (Fig. 9a). PC 1 appeared to separate stressed-samples from their corresponding non-stressed samples (Fig. 9c, d). PC 2 appeared to separate *A. sacrorum* and *A. giraldii* from *A. scoparia* and *A. annua* in both control- and stressed-groups (Fig. 9c), which showed more link with the root morphological traits (Fig. 9c). PC3 including ASA, POD and soluble sugar, classified four species into three groups under drought stress, which are consistent with the types of leaf (Fig. 9d) and indicate the better performance of PC 3 components in isolateral leaf under drought condition.

## Discussion

As a result of long-term natural selection and co-evolution, plants in drought-prone regions have evolved in numerous adaptations to counteract water deficit stress and prevent drought stress damage (Mullet and Whitsitt 1996). *A. scoparia*, *A. sacrorum* and *A. giraldii* could survive on the abandoned land due to their unique adaptive-ability to drought in the Loess hilly region.

### Drought-resistant traits of leaf and root affect the succession of *Artemisia* species on abandoned land

*Artemisia scoparia* as the earliest *Artemisia* species appeared in the abandoned land during the first 1–6 years (Du et al. 2007). In our study, the smaller root system and lower root/shoot ratio were found in *A. scoparia*, which are consistent with the improved soil moisture condition detected in the early period of secondary succession (Du et al. 2007; Jiao et al. 2005; Wang 2003; Wang et al. 2009). These results also indicated that *A. scoparia* allocates more matter and energy to reproductive growth (Fig. 5c), resulting in *A. scoparia* to rapidly occupy resources after the onset of secondary succession, which is consistent with the investigation that *A. scoparia* is annual or biennial species using *r*-strategist reproduction (Du et al. 2006). Although the relatively better soil moisture at the early stage, plants grew on abandoned land was still under water shortage condition. Thus, a not large but succulent root system was a good selection for *A. scoparia* resisting drought during its annual or biennial life cycle. Otherwise, the succulent root is important for *A. scoparia* to cope with accidental drought on abandoned land because 60–70 % of annual precipitation was mainly concentrated in July to September on the Loess Plateau.

However, a large root system was found in *A. sacrorum* and *A. giraldii*, which was advantageous to *A. sacrorum* and *A. giraldii* constantly absorbing water from the deep soil layer, since the shallow layer soil moisture gradually decreased during the succession growing. This change of soil moisture could eliminate the plants with shallow roots, such as *A. scoparia*, resulting in that *A. sacrorum* and *A. giraldii* are widespread in years 9–46 in the abandoned land (Jiao et al. 2008). Additionally, a well-developed xylem was observed in *A. sacrorum* root (Fig. 4a), which may satisfy the water requirements for the relatively high rate of photosynthesis (Fig. 6a). However, *A. sacrorum* may lead to more serious decrease in soil water content, and this condition occurred during the middle stage of secondary succession (roughly 8–30 years). Compared with *A. sacrorum*, the root of *A. giraldii* presents similar root system, but a higher proportion of parenchyma cells and lower proportion of xylem, which indicated that *A. giraldii* root could store more water than *A. sacrorum*. These traits may be in relation to that *A. giraldii* had wider distribution than *A. sacrorum* on the 30–40 year-old abandoned land.

Well-developed leaf trichomes are important for plant adaptation to drought environment because it not only alleviates heavy radiation, but also decreases leaf water loss from transpiration. Our study showed that trichome density was positively correlated with stomatal density (Figs. 1, 2) on the leaves in the three xeromorphic plants, but very few trichomes were found on the *A. annua* leaves. These results indicated that trichomes were important for the growth of three *Artemisia* species on abandoned land and mainly decrease water loss from the stomata. Moreover, the bushy trichome on the adaxial surface of *A. scoparia* leaf could alleviate chloroplast damage from intense light radiation on bare land in the early period of secondary succession.

As the result of drought-adaptation, the leaf anatomy showed that *A. scoparia* and *A. giraldii* contained isolateral leaves with compact mesophyll cells on both abaxial and adaxial surfaces, as well as the stomata distribution on both surfaces of isolateral leaves increasing the flexibility of regulating CO_2_ assimilation and transpiration. Therefore, these two species possibly exhibited the optimal balanced condition between water conservation and CO_2_ assimilation, resulting in relatively increased WUE (Fig. 6c) under drought conditions. The leaf anatomical traits of *A. scoparia* seem to compensate for the weakness of its root morphology, which is significant for this species growing on the abandoned land. More interestingly, *A. sacrorum* presented the highest upper epidermal thickness, cuticle thickness, and compactness, which may strongly compensate for the absence of trichomes on the adaxial surface. However, its transitional form leaf may limit its continuous growth under the low soil moisture condition of the 30–40-year-old abandoned land. By contrast, *A. giraldii* could continuously flourish on the 30–40-year-old abandoned land depending on the superiority of its root and leaf traits. No advantageous leaf traits were found on *A. annua*.

### Water use is an important strategy for *Artemisia* succession on abandoned land

In our study, water consumption assessment of the four *Artemisia* species (Fig. 6b) indicated that plants in the later succession sequence exhibit better regulation of transpiration than that in the early stages, which fulfills the water consumption assessment of native plants and is significant for vegetation restoration, both theoretically and in practice on the Loess Plateau (Chen et al. 2007; Shi and Shao 2000; Guo and Shao 2003). More Interestingly, this result was directly correlated with lower stomatal density (Fig. 2a), which may indicate that stomatal regulation was important for plant adaptation to drought environment, moreover the changes of *Gs* in four species further illustrated this result (Fig. 6d).

Under drought conditions, biomass accumulation can be used to evaluate the drought resistance of plants. In our study, the highest dry weight of *A. sacrorum* among the four *Artemisia* species (Fig. 5b) could be contribute to its transitional form of leaf cells which has high efficiency of CO_2_ exchange and assimilation (Fig. 6a) under well soil moisture condition. Under drought condition, these three dominant species showed relative low growth inhibition, which indicated the better drought-tolerance of *Artemisia* grew on abandoned land.

WUE is an effective parameter used to evaluate the drought resistance of plants. Under control conditions, the relatively high WUE of *A. sacrorum* can be attributed to the perfect cooperation between high root hydraulic supply and leaf structure. However, under drought stress, the high WUE of *A. giraldii* indicated that this species exhibits the optimal strategy to adapt to soil drought of the 30–45 years abandoned land. The decrease in the WUE of *A. sacrorum* under drought conditions could explain why *A. sacrorum* can be a dominant species during the median period of succession. By contrast, the lower WUE of *A. annua* could strongly affect plant survival on the abandoned land.

### The different antioxidants changes in response to drought stress in the four *Artemisia* species further explain their ecological adaptation to soil moisture deficit of abandoned land

A plant antioxidative system is an important mechanism in plants drought-tolerance (Mittler 2002). From our results, the increase in SOD and CAT activity induced by drought exhibited the trend of *A.giraldii* > *A. sacrorum* > *A. scoparia* and *A. annua*. This indicated the importance of induced regulation of SOD and CAT activity in the plants grew at the later stage of succession on the abandoned land. The high APX levels of *A. giraldii* and *A. sacrorum* suggested that a high constitutive APX activity was essential for *Artemisia* survival during the middle or later stage of succession of abandoned land, too. The increase in APX activity induced under drought in *A. scoparia* leaves illustrated that efficient regulation of APX activity was important for *A. scoparia* growth in the early stages of succession on abandoned land. By contrast, the poor regulation of APX activity in *A. annua* leaves results in its poor survival on abandoned land. On the other hand, the increased POX activity in *A. scoparia* and *A. giraldii*, as well as the decreased POX activity in *A. sacrorum* (25 %) and *A. annua* (39 %), seem to associate with their types of leaf cells (Fig. 9b, d). In summary, these results suggested that the different ant-oxidative enzymes activity in four *Artemisia* species is also influence their growth on the abandoned land.

The function of proline in cellular homeostasis, as described by Szabados and Savoure (2010), includes redox balance and energy status, osmoprotection, signaling, and trigger for specific gene expression. Based on our results, the proline content of the four *Artemisia* species may reflect the degree of drought stress. Soluble sugar which is a class of osmolytes, exhibits strong responses to drought stress (Seki et al. 2007). ASA is an important water-soluble metabolite that functions as antioxidant and protects carotenoid, glutathione, or tocopherols from oxidation reaction (Asensi-Fabado and Munné-Boschemail 2010; Mittler 2002). In our research, the increased levels of these two soluble metabolites in the leaves of the four *Artemisia* plants followed the order of *A. scoparia* and *A. giraldii* > *A. sacrorum* > *A. annua*; these decreased levels seem to associate with the leaf types of each species (Fig. 9b, d). This results suggested that the stronger drought-adaptation in three dominant species than that in *A. annua*.

Compared with ASA, carotenoid is a class of lipid solutes as antioxidant in plastids, especially in single oxygen levels within thylakoid membranes (Mittler 2002). According our investigation, carotenoid contents followed the order of *A. sacrorum* (transitional form) and *A. annua* (bifacial leaves) > *A. scoparia* and *A. giraldii* (both isolateral leaves) under control conditions. However, the increase in carotenoid content showed that *A. scoparia* and *A. giraldii* (both isolateral leaves) > *A. sacrorum* (transitional form) and *A. annua* (both bifacial leaves) under drought conditions. This result reflected that the more increased Car content in leaf of *A. scoparia* and *A. giraldii* was important for their growth on abandoned land.

MDA content can reflect the degree of lipid peroxidation in the cell and indirectly reflect the degree of damage to the cell membrane system by ROS or free radicals (Mittler 2002). In our study, *A. sacrorum* and *A. giraldii* showed higher MDA content than *A. scoparia* and *A. annua* (Figs. 7a) in both control and drought stress groups. This result was strongly correlated with the root morphology (Fig. 9a, c). We thought that the root system with long and many branch roots (i.e., developed roots) exhibit higher potential risk that the part of roots is in less moisture soil. Consequently, abscisic acid (ABA) level may easily increase in the large root system, leading to high levels of ABA in leaves, thereby increasing ROS levels (Jiang and Zhang 2002). However, *A. scoparia* and *A. annua* roots may generate less ABA because of less lateral roots, which go against to resist drought.

## Conclusion

On the Loess Plateau, the high soil moisture content detected in the early stages of the succession of abandoned land is advantageous for growth of *A. scoparia*. In addition, *A. scoparia* had a low root/shoot ratio which indicated more materials and energy to reproductive growth and rapidly acquire numerous resources in the early period of succession. The bushy trichomes distributed on the *A. scoparia* leaf could prevent excess light and transpiration during the early stage of succession. Moreover, regulations of APX, soluble sugars, and carotenoid, as well as control of POX, proline, and ASA levels, are important for *A. scoparia* growth on abandoned land. When succession continued, the shallow soil moisture of the abandoned land decreased, but *A. sacrorum* grew because of its developed root system and drought-resistant leaf anatomical traits. However, the performances of water using characteristic in *A. sacrorum* may limit its further reproduction on the abandoned land in the later stages of succession, where soil moisture is low. *A. giraldii* could combine the superior traits of the two former species, such as a developed root system and xeromorphic anatomical traits of isolateral leaf. Additionally, *A. giraldii* exhibited a flexible regulation between transpiration and CO_2_ assimilation under drought conditions. Moreover, *A. giraldii* optimally regulated antioxidative enzymes and antioxidants among the four *Artemisia* species. Based on these traits, *A. giraldii* continuously grow in the later stages of succession. *A. annua* does not present any morphological or anatomical drought-resistant traits. Compared with the other three *Artemisia* species, the performances of antioxidative enzymes, soluble sugar, and ASA were unsatisfactory under drought conditions. Although the levels of proline and carotenoid accumulation in leaves were high, the dry weight and WUE decreased under drought conditions. These results implied that *A. annua* would not survive on abandoned land.
